# As warm as competent, really? On the importance of stereotype facets in the context of autism

**DOI:** 10.1111/bjso.70112

**Published:** 2026-07-08

**Authors:** Camille Sanrey, Vincent Yzerbyt

**Affiliations:** ^1^ Université de Strasbourg, Laboratoire de Psychologie Des Cognitions Strasbourg France; ^2^ Université Catholique de Louvain, Laboratoire SOCLAB Ottignies‐Louvain‐la‐Neuve Belgium

**Keywords:** autism, big two, facets of social evaluation, stereotypes

## Abstract

Previous work using the Stereotype Content Model reveals that autistic individuals come across as equally warm, if not warmer, than competent. This pattern runs against the widespread representations of autistic individuals as highly intelligent but lacking in social skills. The present research addresses this inconsistency by considering the facets of social judgement. Across two studies (Study 1—students' sample: *N* = 497, Study 2—general population sample: *N* = 667), participants completed trait attributions and reported spontaneous stereotypes. Results consistently revealed that respondents perceived autistic individuals as low in friendliness but high in morality and higher in ability than in assertiveness. Moreover, whereas friendliness and ability dominated spontaneous descriptions, morality and assertiveness were evoked only marginally. These findings highlight the added value of analysing stereotypes at the facet level, at least when it comes to autistic individuals, and offer a more nuanced understanding of stereotypic representations of this group.

## INTRODUCTION

What if somebody told you that autistic individuals are just as warm (e.g., sociable) as they are competent (e.g., intelligent)? This information likely contradicts your view, and you would not be alone in reacting this way. Indeed, popular representations of autism often portray autistic individuals as highly intelligent while lacking social skills (Draaisma, 2009). Interestingly, however, recent work conducted within the Stereotype Content Model (SCM; Fiske et al., 2002) framework suggests that people perceive autistic individuals as either equally warm and competent or warmer than competent (Aubé et al., 2023; Canton et al., 2023). Gaining a better understanding of these inconsistencies seems important to shed light on specific inclusion difficulties, as stereotypes predict emotional and behavioural responses (Cuddy et al., 2008; Koch et al., 2024). The present research explores this apparent inconsistency by analysing stereotypes associated with autistic individuals at a higher level of resolution. Moreover, we rely on two complementary measures of stereotypes. First, building on recent theoretical and empirical developments, we analyse stereotypic representations of autistic individuals in terms of the so‐called facets of social evaluation (Abele et al., 2021; Yzerbyt et al., 2025) rather than at the level of the two fundamental dimensions. Second, in contrast to most studies that rely only on trait attribution tasks to tap stereotypes, we further our understanding by turning to spontaneous measures of the relevant stereotypes (Nicolas, 2023; Nicolas et al., 2021).

### A general theoretical framework for studying stereotypes

For more than two decades, the SCM has been the dominant model when it comes to studying stereotypes of social groups. Building on a long tradition of work within the impression formation literature and the research on intergroup relations, Fiske and her colleagues (Fiske et al., 1999, 2002) proposed that individuals organize their perceptions along two primary dimensions often referred to as the Big Two. The dimension of warmth (e.g., ‘friendly’, ‘sociable’, ‘sincere’), currently labelled the horizontal dimension, refers to the perception of people's intentions, helping perceivers determine whether a group is likely to be an ally or a threat. The dimension of competence (e.g., ‘intelligent’), now known as the vertical dimension, denotes the perceived ability of a group and its members to enact those intentions (Fiske et al., 2002; for recent reviews, Abele et al., 2021; Yzerbyt et al., 2025).

The SCM work on autism mainly relied on traits attributions and suggests that people perceive autistic people either as ‘warmer than competent’ (Aubé et al., 2023; Görzig et al., 2020) or at least as ‘as warm as competent’ (Boysen et al., 2020; Canton et al., 2023; but see Schell et al., 2024 for an exception within the school context). These findings appear somewhat surprising when contrasted with data emerging from work on the social representations of autism. Indeed, using ecological data collection tools, these efforts reveal an image of autistic people as highly intelligent (i.e., savantism, Draaisma, 2009) but lacking in social skills (Wood & Freeth, 2016; see Mittmann et al., 2024 for a review of media representation). Reconciling these divergent messages is particularly important. Indeed, besides its aim to investigate the content of stereotypes, SCM work has an interest in the consequences of social evaluation. Specifically, the stereotype attached to a given target group is expected to trigger specific emotional and behavioural reactions (Cuddy et al., 2008). Groups associated with higher warmth than competence, that is, falling into what the SCM calls the ‘paternalistic quadrant’, are expected to be mainly associated with reactions of pity and to lead to active facilitation (help, inclusion) or passive harm (avoidance). In contrast, groups associated with higher competence than warmth, that is, falling in the so‐called ‘envious’ quadrant, are presumably associated with reactions of jealousy and lead to passive facilitation (cooperation) or active harm (fighting against). Hence, analysing the perception of autistic individuals should help understanding specific difficulties that they may encounter in everyday life. The key question is thus to reconcile the divergent messages that emanate from the SCM research on the one hand and from the social representation efforts on the other? Here, we would like to argue that this inconsistency can be resolved by adopting two complementary approaches. First, we suggest that it is necessary to analyse the content of stereotypes by examining the four facets rather than the more global two dimensions of social evaluation (Abele et al., 2021; Yzerbyt et al., 2025). Second, we propose that, rather than using self‐report scales, we may also want to dig into people's spontaneous representations, allowing access to more specific and ecologically sound information (Nicolas, 2023; Nicolas et al., 2021).

### The added value of the four facets of social evaluation

Theoretical and empirical efforts on the Big Two in the context of an adversarial collaboration (Ellemers et al., 2020) have highlighted the value of distinguishing four facets, two per dimension (Abele et al., 2021; Koch et al., 2021; for a review, Yzerbyt et al., 2025). While the horizontal dimension comprises the facets of friendliness (e.g. ‘warm’) and morality (e.g. ‘honest’), the vertical dimension includes the facets of ability (e.g. ‘intelligent’) and assertiveness (e.g. ‘self‐confident’; Abele et al., 2021). Recent work shows the added value of the four facets as compared to simply looking at the two dimensions when studying people's perception of a variety of groups (Barbedor et al., 2024). Taking these facets into consideration allows for a more nuanced understanding of stereotyping and its consequences, as each facet predicts specific behavioural responses. Indeed, Koch et al. (2024) showed that each facet orients distinct behavioural reactions in the context of economic games. With respect to horizontal facets, friendliness drives behaviours in a loyalty game, whereas morality predicts behaviours for a deception game. Considering vertical facets, ability shapes behaviours within investment game, whereas assertiveness is the relevant facet within an hubris game.

Other work showed that each facet produces specific impressions. Regarding the horizontal dimension, morality translates into helping intentions more than friendliness (Brambilla et al., 2013). At the same time, friendliness is mainly associated with pleasure and willingness to interact, while morality is associated with quietness and the absence of threat (Carrier et al., 2014). Turning to the vertical dimension, assertiveness is more tightly related to status perception than ability (Carrier et al., 2014; Yzerbyt et al., 2025). As a result, assertiveness plays a greater role than ability when considering access to high status positions in society, with perceptions of low assertiveness even being a potential barrier.

The above research emphasizes the interest in considering the four facets of social evaluation to better grasp the complexity of stereotypes and their potential consequences. Building on these efforts, we propose that much insight can be gained by looking at people's perceptions of autistic individuals using the four facets rather than simply the two dimensions. Regarding the horizontal facets, autism is predominantly associated with perceived deficits in social skills (Wood & Freeth, 2016), which translates into low levels of friendliness. At the same time, autistic individuals are often seen a possessing a form of ‘moral superiority’ (Draaisma, 2009), notably due to a presumed inability to lie (Baron‐Cohen, 2008). In short, the horizontal facets should reveal the concurrent presence of low friendliness and high morality. With respect to the vertical facets, the notion of ‘savantism’ often attributed to autistic individuals (Draaisma, 2009; Wood & Freeth, 2016) clearly relates to high ability. At the same time, some studies suggest perceptions of low assertiveness (e.g. low self‐confidence ratings, Schell et al., 2024), although information regarding assertiveness remains scarce.

### The added value of spontaneous stereotypes

Classical approaches using trait attributions come with two main limitations. First, most SCM studies only use positive traits to lower social desirability concerns (Aubé et al., 2023; Canton et al., 2023; Fiske et al., 2002). This feature may be ill‐adapted to get a hold on stereotypes about stigmatized groups (Sanrey et al., 2026). Second, trait attributions force people to position themselves on preselected traits, which may fail to access dimensions or facets that are spontaneously associated with the groups (Nicolas et al., 2021). That is, consciously reflecting on some a priori list of traits may be different from spontaneously associating traits to a group. The use of an ‘unobtrusive and unconstrained approach to measurement’ (Nicolas et al., 2021, p.179) likely offers an ecological and relevant alternative to study stereotypes associated with social groups (Nicolas, 2023). Moreover, the analysis of spontaneous productions is greatly facilitated by the recent development of stereotype dictionaries (Nicolas et al., 2021), which include the four facets among other stereotype dimensions (e.g., status, beliefs, emotion).

Recent work on spontaneous stereotypes demonstrated the soundness of this type of measure to access stereotypes towards various groups, confirming that the four facets are the most prevalent dimensions (Nicolas, 2023). Interestingly, although the four facets prevail in spontaneous descriptions of social groups, a substantial degree of variability also emerges depending on the targets. For example, Nicolas (2023) found that morality and friendliness seem to be the most prevalent facets when evaluating social groups (e.g. ‘homeless’, ‘vegans’, ‘CEO’), whereas assessments of facial expressions rely primarily on friendliness traits (Nicolas et al., 2021). This heterogeneity also shows in recent work focusing on spontaneous stereotypes of mental illnesses (Sanrey et al., 2026). For example, while phobia was mainly associated with assertiveness traits, schizophrenia was mostly associated with both morality and friendliness traits. Such findings highlight the sensitivity of spontaneous measurement in accessing stereotypical beliefs.

Building on these efforts, we assumed that measuring spontaneous stereotypes may provide useful additional information on top of the SCM trait attributions. For example, stereotypes about autistic individuals' lack of social skills may manifest both through low attributions of friendliness on the trait attributions and spontaneous associations of negative friendliness.

## OVERVIEW OF THE RESEARCH

In two studies, we presented participants with SCM traits attributions and hypothesized that, within the horizontal dimension, friendliness would be rated significantly lower than morality (H1) while, within the vertical dimension, ability would be rated higher than assertiveness (H2). We also collected spontaneous stereotypes to assess the prevalence of the different facets in unconstrained descriptions. We predicted that friendliness (H3) and ability (H4) would be more prevalent than morality and assertiveness, respectively.

The two studies used the same methodology to examine two different French samples and were approved by the University of Strasbourg ethical board (Unistra/CER/2024‐03). The first study consisted of a sample of first‐ and second‐year psychology students, a population that can be expected to be somewhat educated about stereotypes and autism. The sample of the second study consisted of respondents from the general population recruited through social media. We report on how we determined our sample size, all data exclusions and all measures in the two studies.

All datasets, codebooks and analysis scripts can be found on the OSF page (Study 1: https://osf.io/t8qcm/?view_only=255c76e04e0049dcbbefeb00e7be042f; Study 2: https://osf.io/wp89y/?view_only=1abc9a9dc51a4f3d9fde50a1706d27be). We analysed our data using R 4.3.1 (R Core Team, 2023). Although initially pre‐registered for different research aims, the evolution of our research objectives led us to present this work as exploratory. The data available on OSF include data on other measures that are not directly relevant to the objectives of this paper and are therefore not presented here.

## STUDY 1

### Participants

We recruited a total of 503 participants during first‐ and second‐year psychology courses in a French University. We excluded six participants due to age (under 18), resulting in a final sample of 497 participants (424 women, 64 men, 9 non‐binary people, M_age_ = 19.89, SD_age_ = 3.44, min = 18, max = 50). A sensitivity analyses conducted using G*Power (Faul et al., 2007) revealed that this sample size allows for the detection of a small effect size (*d* = 0.12, *f* = 0.06) with four measurements (i.e. the four facets) and 90% power.

### Materials and methods

#### Spontaneous stereotypes

Participants were to list up to five characteristics that they believed ‘people in general’ most strongly associate with autistic individuals. They had to do so using single words (two words if necessary; see Nicolas et al., 2021 for a comparable procedure). Hence, participants learned that they did not need to personally endorse the listed characteristics. We used this societal format based on previous SCM work to reduce social desirability while still providing meaningful insights into culturally shared representations (e.g. Fiske et al., 2002).

#### Trait attributions

Using the same societal instructions as for spontaneous stereotypes, participants indicated the extent to which they believed ‘people in general’ attributed 12 traits commonly used in the SCM literature to autistic individuals, using scales ranging from 1 (not at all) to 9 (totally). Traits were as follows: ‘sociable’, ‘friendly’ and ‘warm’ for friendliness, ‘moral’, ‘sincere’ and ‘honest’ for morality, ‘capable’, ‘intelligent’ and ‘efficient’ for ability and ‘persevering’, ‘determined’ and ‘self‐confident’ for assertiveness. Confirmatory factorial analyses confirmed that the fit of the four facet model (CFI = .93, TLI = .90, RMSEA = .08, SRMR = .07, BIC = 23116.79) was better than for the two‐dimension model (CFI = .66, TLI = .57, RMSEA = .17, SRMR = .14, BIC = 23688.07), Δ*χ* ^2^ = 602.32, *p* < .001, or three‐facet model (CFI = .87, TLI = .83, RMSEA = .11, SRMR = .07, BIC = 23233.60), Δ*χ* ^2^ = 135.44, *p* < .001. Omega computed based on the Confirmatory Factorial Analysis (CFA) supports the reliability of each of the four facets: friendliness (*ω* = .81), morality (*ω* = .76), ability (*ω* = .78) and assertiveness (*ω* = .76).

#### Attention checks and sociodemographic variables

We included three attentional checks in the questionnaire. One multiple‐choice question after the consent form asked participants to select the theme of the study (i.e. ‘the perception of autistic people’; 96.18% of success), and two items within the scales asked them to select either the number ‘3’ (82.09% of success) or the number ‘2’ (88.53% of success). Participants also reported their gender, age and level of education. In addition, they responded to three items assessing their familiarity, perceived knowledge and contact with autistic individuals on scales ranging from 1 to 9, allowing us to compute a familiarity index (*α* = .84, M = 4.08, SD = 1.87).

## RESULTS

Following recent recommendations, we used a more conservative Type I error level for the analyses of trait attributions (Maier & Lakens, 2022). Using Lakens' shinyapp (https://shiny.ieis.tue.nl/JustifyAlpha/), we computed the adjusted alpha level for a two‐sided paired *t*‐test (i.e. comparison between the two fundamental dimensions), with *N* = 497, Cauchy scale of 0.707 (default) and a BF of 3 (i.e. H1 should be 3 times higher than H0 to reject H0). This choice was based on practical recommendations and in light of the exploratory nature of this study, which suggest the use of a conservative choice (i.e., BF 3) to obtain at least moderate evidence in favour of H1 if the *p*‐value is significant. Accordingly, we considered the results regarding trait attribution to be statistically significant at *p* < .004 (instead of.05).

Considering the exploratory nature of this study, we also aimed to conduct complementary analyses aiming to maximize the robustness of the findings (see Supporting Information for results concerning these robustness tests). Here, we wanted to ensure that the observed effects were stable across three different variations. First, we rerun the analyses with the familiarity index as a covariate. Second, all models were computed again on the subsample of participants who responded correctly to all attention checks. This led to a subsample of *n* = 391, after the exclusion of 106 participants (i.e., 21.33%) who failed at least one attention check. Finally, we relied on three traits per facet classically used in the literature. However, Koch et al. (2024) recently validated a two‐item per facet. This led us to rerun our analyses with this shorter measure to check for the robustness of our conclusions.

### Traits attributions

**FIGURE 1:**
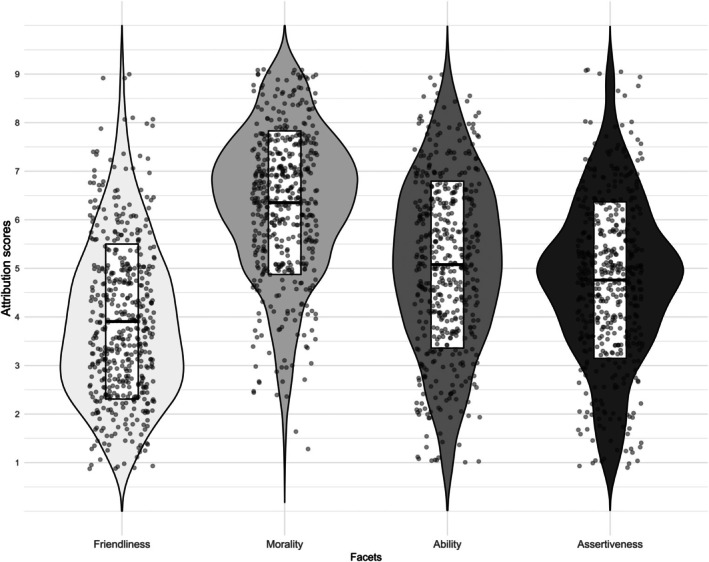
Violin plot of facets' attribution scores (Study 1).

Confirming previous studies based on the SCM, participants perceived autistic people as slightly warmer (M = 5.13, SD = 1.19) than competent (M = 4.92, SD = 1.46), *t*(496) = 3.24, *p* = .001, *d* = 0.15, 95% CI [0.06, 0.23]. As hypothesized, the facets scores differed significantly, *F*(2.69, 1335.90) = 290.73, *p* < .001, partial *η* ^2^ = .37 (see Table 1 and Figure 1). Bonferroni‐corrected post hoc comparisons supported our hypotheses in that participants attributed significantly less friendliness than morality (H1), *t*(469) = −27.97, *p* < .001, *d* = −1.25, and significantly less assertiveness than ability (H2), *t*(469) = 4.43, *p* < .001, *d* = 0.20.

**TABLE 1:** Means (standard deviations) and correlations [95% CI] between facets attribution (Study 1).

	Mean (SD)	1	2	3	4
1. Friendliness	3.90 (1.60)	–			
2. Morality	6.35 (1.48)	.20 *** [.11–.28]	–		
3. Ability	5.08 (1.72)	.11 * [.03–.20]	.39 *** [.31–.46]	–	
4. Assertiveness	4.76 (1.61)	.23 *** [.14–.31]	.43 *** [.36–.50]	.54 *** [.48–.60]	–

* *p* < .05.

*** *p* < .001.

### Spontaneous stereotypes

We analysed participants' spontaneous stereotypes using the SADCAT package (Nicolas et al., 2021). This tool consists of stereotype dictionaries which automatically code linguistic productions based on their representativeness (1 = coded, 0 = non‐coded) of specific dimensions, including the four facets. Moreover, the package codes for binary valence (negative vs. positive), based on information extracted from pre‐existing databases (see Nicolas et al., 2021 for details). Of importance, these dictionaries have been validated to gauge spontaneous stereotypes across various contexts (Nicolas et al., 2021; Nicolas, 2023; Sanrey et al., 2026).

Participants produced a total of 1589 characteristics, of which 1116 (70.23%) fell in at least one of the four facets of interest. Although the dictionaries allow for multiple coding (i.e. a single characteristic may be coded into more than one facet), we focused on pure characteristics—that is, those coded in one facet only—yielding 1052 coded characteristics (66.21% of the overall production).

Among these 1052 pure characteristics, 420 pointed to friendliness (26.4%), 62 to morality (3.90%), 443 to ability (27.9%) and 127 to assertiveness (7.99%). Because participants could provide more than one response pertaining to more than one facet, we could not rely on a standard contingency table to compute chi‐square statistics. Instead, we turned to Bayesian analyses which we conducted in R (Version 4.6.1) using the **brms** package to fit multilevel models for the four facet categories. The 1052 responses were modelled using weakly informative priors, and parameters were estimated via Hamiltonian Monte Carlo sampling implemented in Stan. Four chains were run with sufficient warm‐up iterations, and convergence was assessed using R‐hat values and visual inspection of trace plots. A Bayesian categorical logistic model was fitted to the full dataset (*N* = 1052) with participant as a random intercept. Ability served as the reference category. The posterior estimates indicated that Assertiveness (12.07%, *β* = −1.31, 95% CI [−1.58, −1.09]) and Morality (5.93%, *β* = −2.08, 95% CI [−2.52, −1.76]) were substantially less likely to be selected than Ability, whereas Friendliness (39.93%) did not differ meaningfully from Ability (42.06%, *β* = −0.06, 95% CI [−0.20, 0.08]). Random‐intercept variability was moderate for Assertiveness (SD = 0.33, 95% CI [0.02, 0.81]) and Morality (SD = 0.43, 95% CI [0.02, 1.11]) and smaller for Friendliness (SD = 0.16, 95% CI [0.01, 0.43]).

**FIGURE 2:**
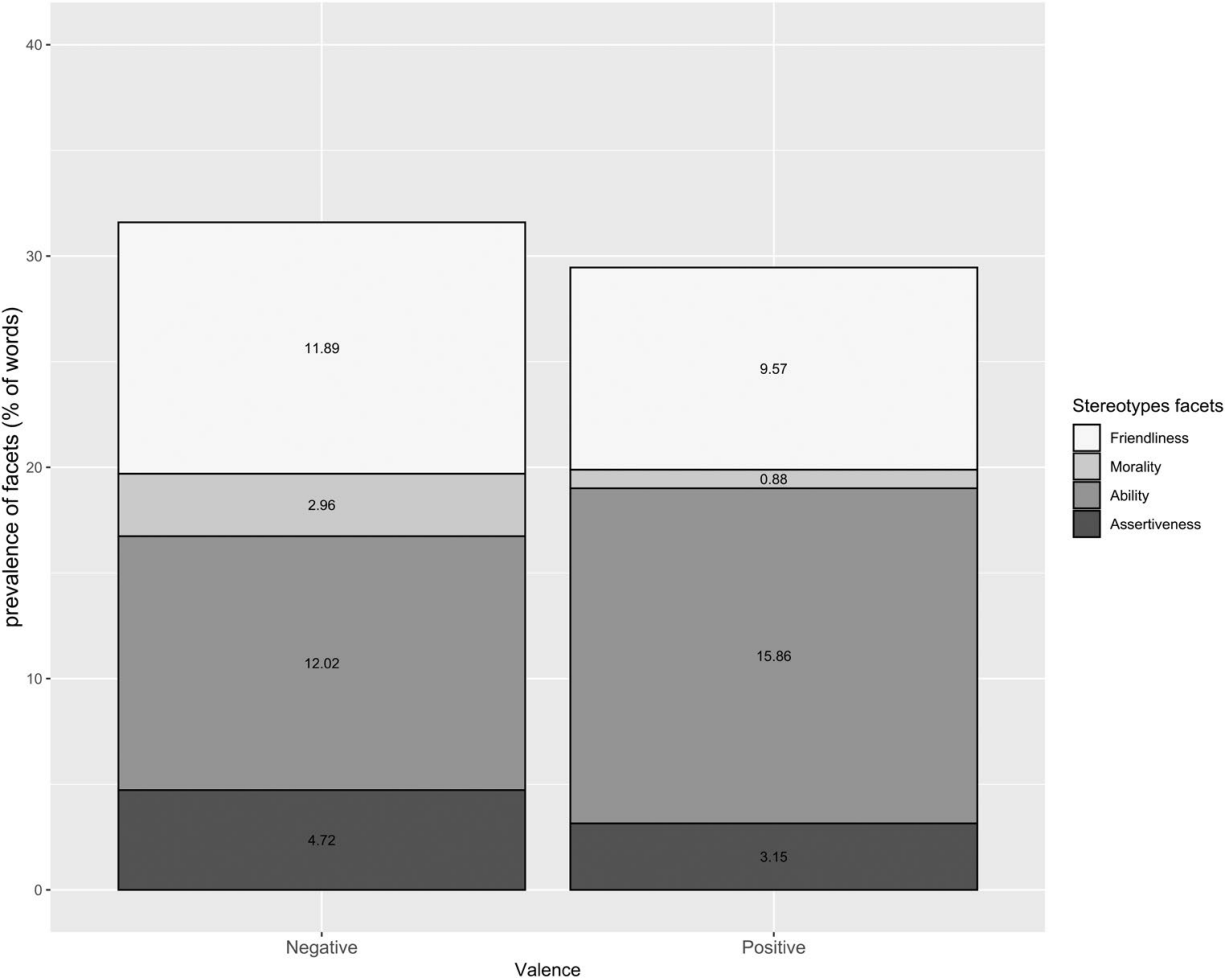
Prevalence of each facet in spontaneous production according to valence (Study 1).

We also coded the characteristics for binary valence (i.e. positive vs. negative, see Figure 2). A Bayesian multilevel logistic model was fitted to the ability data. The probability of observing positive characteristics for ability was significantly above chance (60%, posterior mean intercept = 0.41, 95% CI [0.14, 0.71]). The model also indicated substantial between‐participants variability, with a random‐intercept standard deviation of 1.08 (95% CI [0.30, 1.84]). Regarding assertiveness, the probability of finding positive characteristics was significantly below chance (39%; posterior mean intercept = −0.46, 95% CI [−0.92, −0.07]). Here too, the between‐participants variability was rather large, with a random‐intercept standard deviation of 0.60 (95% CI [0.03, 1.77]). Turning to friendliness, the data revealed a significantly lower probability of finding positive (44%, posterior mean intercept = −0.23, 95% CI [−0.46, −0.01]) as opposed to negative characteristics. Again, there was between‐participants variability, with a random‐intercept standard deviation of 0.29 (95% CI [0.01, 0.81]). Finally, for morality, there was also a significantly lower probability of observing positive (7%, posterior mean intercept = −0.23, 95% CI [−0.46, −0.01]) compared to negative morality characteristics. Not surprisingly, we observed extreme between‐participants variability, with a random‐intercept standard deviation of 3.17 (95% CI [0.22, 8.61]).

### Discussion

In Study 1, we examined psychology students' stereotypes about autistic individuals. We not only relied on the four facets of social evaluation rather than the classical two dimensions, but we also added a measure of spontaneous stereotypes to the more traditional trait attributions. With these two improvements, we hoped to shed light on apparent inconsistencies between the results reported in the SCM literature and those emerging from the more general literature dealing with social representations.

First, results on trait attributions confirmed that, although the societal perceptions of psychology students suggest that autistic individuals are associated with higher warmth than competence, a closer examination at the finer grained level of facets reveals important differences between the facets pertaining to each dimension. Considering the horizontal dimension, the representations of autistic individuals show them to be less friendly than moral. This difference is consistent with social representations depicting autistic individuals as lacking in social skills, but as having high morality, notably through the presumed inability to lie (Baron‐Cohen, 2008; Draaisma, 2009). Turning to the vertical dimension, respondents saw autistic individuals as more able than assertive. Again, this pattern is consistent with social representations highlighting ‘savantism’ but low self‐confidence (Schell et al., 2024).

Turning to respondents' spontaneous productions, the facets of friendliness and ability were the most prevalent. Confirming the pattern of trait attributions, friendliness was associated with more negative than positive characteristics while ability was associated with more positive than negative characteristics. The two other facets, namely morality and assertiveness, only emerged minimally in spontaneous productions. These findings show the benefit of combining trait attributions and spontaneous productions in that they allow for a globally convergent yet complementary picture. While the two measures reveal similar patterns regarding friendliness, ability and assertiveness, results regarding morality deserve special comment. Indeed, the trait attributions were aligned with the view often encountered in the literature regarding the moral superiority of autistic individuals. Still, spontaneous measures reveal a more ambivalent picture. Only 3.90% of the characteristics spontaneously listed by participants reflect morality, with a majority of these being negative. Negative morality characteristics are mainly related to perceptions of potential violence, which aligns with a popular view associating autism and violent behaviours (Maras et al., 2015). In sum, when asked to react with respect to a set of preselected traits, participants pointed to the moral superiority of autistic individuals, while their spontaneous representations point to a much lesser extent to this same facet, and when they do, the reference is decidedly more one of violence and negativity.

Globally, the present data provide encouraging support for our hypotheses. However, the fact that we relied on a convenience sample of psychology students commands some degree of caution. Indeed, psychology students are likely to be somewhat knowledgeable about autism, a characteristic that prevents us from generalizing the present findings to the general population. To further establish the robustness of the obtained pattern, we conducted a second study using the exact same methodology but relying this time on a sample issued from the general population.

## STUDY 2

### Participants

We recruited 672 French participants via social media. We excluded five participants due to age (under 18), resulting in a final sample of 667 participants (364 women, 264 men, 37 non‐binary people and 2 who did not indicate their gender, M_age_ = 34.54, SD_age_ = 10.27, min = 18, max = 74). A sensitivity analysis conducted using G*Power (Faul et al., 2007) indicated that this sample size allows for the detection of a small effect size (*d* = 0.10, *f* = 0.05) with four measurements (i.e. the four facets) and 90% power. Participants did not receive financial compensation but secured participation in a lottery for a 100€ gift box.

### Materials and methods

We used the same materials and procedure as for Study 1.

#### Spontaneous stereotypes

Again, participants were to list up to five characteristics that they believed people in general associate most strongly with autistic individuals, using single words (two words if really necessary).

#### Trait attributions

Participants indicated the extent to which they believed people in general attribute the same 12 traits using scales ranging from 1 (not at all) to 9 (totally), depicting the four facets: friendliness, morality, ability and assertiveness. As for Study 1, confirmatory factor analyses confirmed that the fit of the four facet model (CFI = .93, TLI = .90, RMSEA = .07, SRMR = .05, BIC = 31617.63) was better than for the two‐dimension model (CFI = .72, TLI = .65, RMSEA = .12, SRMR = .09, BIC = 31992.79), Δ*χ* ^2^ = 407.67, *p* < .001 and three‐facet model (CFI = .91, TLI = .88, RMSEA = .07, SRMR = .06, BIC = 31644.74), Δ*χ* ^2^ = 46.62, *p* < .001. Omega computed based on the CFA indicated that the reliability of each of the four facets was acceptable: friendliness (*ω* = .73), morality (*ω* = .74), ability (*ω* = .63) and assertiveness (*ω* = .68).

#### Attention checks and sociodemographic variables

We included the same three attentional checks as in Study 1 in the questionnaire (check 1: 99.85% of success; check 2: 82.61% of success; check 3: 88.16% of success). Participants also reported their gender, age and level of education and responded to the same three items assessing their familiarity, perceived knowledge and contact with autistic individuals on scales ranging from 1 to 9 (familiarity index: *α* = .88, M = 5.02, SD = 2.03).

## RESULTS

As for Study 1, we use a more conservative Type I error level. Using Lakens' shinyapp, we computed the adjusted alpha level for a two‐sided paired *t*‐test with *N* = 650 and a BF of 3. Accordingly, we considered results as statistically significant at *p* < .001 (instead of .05). Again, to maximize the robustness of the findings, we conducted complementary analyses on the same three variations as in Study 1 (see Supporting Information for results concerning these robustness tests).

### Traits attributions

**FIGURE 3:**
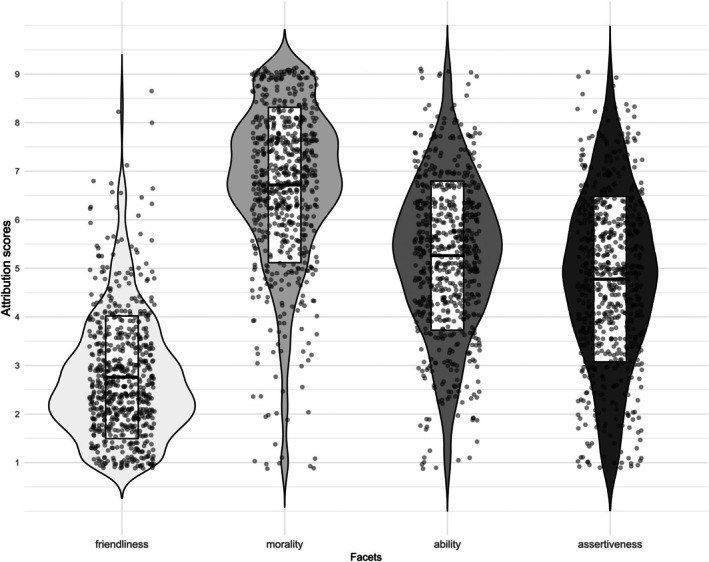
Violin plot of facets' attribution scores (Study 2).

This time, participants perceived autistic people as slightly less warm (M = 4.74, SD = 1.12) than competent (M = 5.02, SD = 1.41), *t*(666) = −5.29, *p* < .001, *d* = −0.20, 95% CI [−0.28, −0.13]. This difference at the dimension level notwithstanding, the same general pattern as in Study 1 emerged at the facet level, *F*(2.92, 1943.67) = 1111.74, *p* < .001, partial *η* ^2^ = .63 (see Table 2 and Figure 3). Bonferroni‐corrected post hoc comparisons again supported our hypotheses: participants attributed significantly less friendliness than morality (H1), *t*(666) = −56.63, *p* < .001, *d* = −2.19 and significantly less assertiveness than ability (H2), *t*(666) = 7.80, *p* < .001, *d* = 0.30. As a comparison between the means in Tables 1 and 2 reveals, Study 2 participants expressed somewhat harsher friendliness judgements than the students taking part in Study 1.

**TABLE 2:** Means (standard deviations) and correlations [95% CI] between facets attribution (Study 2).

	Mean (SD)	1	2	3	4
1. Friendliness	2.76 (1.26)	–			
2. Morality	6.72 (1.60)	.22 *** [.15–.29]	–		
3. Ability	5.26 (1.53)	.19 *** [.11–.26]	.35 *** [.28–.41]	–	
4. Assertiveness	4.78 (1.70)	.23 *** [.16–.30]	.35 *** [.29–.42]	.51 *** [.46–.57]	–

*** *p* < .001.

We conducted an integrative analysis to compare the scores obtained in Study 1 (with psychology students) with those obtained in Study 2 (with the general population). More specifically, we wanted to identify any differences regarding facet attribution, to shed light on the inconsistency at the dimension level. We submitted the data to a mixed model ANOVA using facet as a within‐participant variable and study as a between‐participants variable. The facet by study interaction was significant, *F*(2.87, 3339.58) = 79.43, *p* < .001, partial *η* ^2^ = .06. A series of contrasts targeting differences between Study 1 and Study 2 for each facet revealed that psychology students (Study 1) attributed higher friendliness (M = 3.90, SD = 1.60) than members of the general population did (Study 2; M = 2.76, SD = 1.26), *t*(1162) = 13.69, *p* < .001, but slightly lower morality (M_Study 1_ = 6.35, SD_Study 1_ = 1.48; M_Study 2_ = 6.72, SD_Study 2_ = 1.60), *t*(1162) = −3.96, *p* < .001. Interestingly, we found no significant differences between the two samples for ability, *p* = .054, nor for assertiveness, *p* = .849. In sum, students seem less inclined to question the presence of friendliness than participants from the general population, while being also a little less positive regarding morality.

### Spontaneous stereotypes

As in Study 1, we analysed spontaneous stereotypes using the SADCAT package (Nicolas et al., 2021) which automatically codes linguistic productions based on their representativeness (1 = coded, 0 = non‐coded) of each facet. They produced a total of 2399 characteristics, of which 1543 (64.32%) fell in at least one facet of interest. Among these 1543 characteristics, 1486 (61.94% of the overall production) are ‘pure’ characteristics (i.e. characteristics coded in only one facet).

Among these pure characteristics, 759 pointed to friendliness (31.6%), 91 to morality (3.79%), 482 to ability (20.1%) and 154 to assertiveness (6.42%). As in Study 1, we relied on Bayesian analyses which we conducted in R (Version 4.6.1) using the **brms** package to fit multilevel models for the four facet categories. A Bayesian categorical logistic model was fitted to the full dataset (*N* = 1486) with participant as a random intercept. Ability served as the reference category. The posterior estimates indicated that Assertiveness (10.41%, *β* = −1.19, 95% CI [−1.43, −0.98]) and Morality (6.14%, *β* = −1.77, 95% CI [−2.15, −1.50]) were substantially less likely to be selected than Ability, whereas Friendliness (51.05%) was significantly more likely to emerge than Ability (32.40%, *β* = 0.46, 95% CI [0.34, 0.57]). Random‐intercept variability was moderate for Assertiveness (SD = 0.28, 95% CI [0.01, 0.72]) and Morality (SD = 0.43, 95% CI [0.02, 1.01]) and smaller for Friendliness (SD = 0.09, 95% CI [0.00, 0.27]).

**FIGURE 4:**
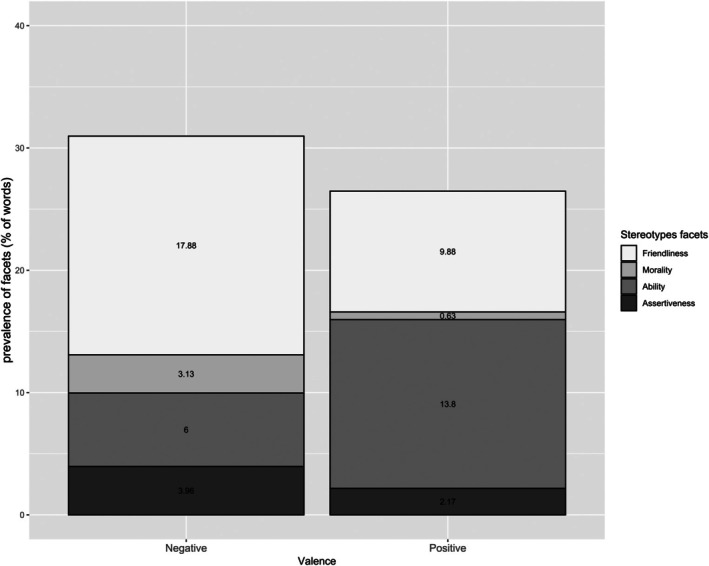
Prevalence of each facet in spontaneous production according to valence (Study 2).

As in Study 1, we coded the characteristics for binary valence (i.e. positive vs. negative, see Figure 4). A Bayesian multilevel logistic model was fitted to the ability data. Again, the probability of observing positive characteristics for ability was very significantly above chance (71%, posterior mean intercept = 0.87, 95% CI [0.64, 1.11]). The model also indicated substantial between‐participants variability, with a random‐intercept standard deviation of 0.31 (95% CI [0.01, 0.80]). Regarding assertiveness, the probability of finding positive characteristics was significantly below chance (33%, posterior mean intercept = −0.71, 95% CI [−1.26, −0.29]), with important between‐participants variability, with a random‐intercept standard deviation of 0.74 (95% CI [0.03, 2.26]). Turning to friendliness, the data showed a significantly lower probability of finding positive characteristics (35%, posterior mean intercept = −0.62, 95% CI [−0.81, −0.45]) as opposed to negativeones. Again, there was sizeable between‐participants variability, with a random‐intercept standard deviation of 0.37 (95% CI [0.02, 0.89]). Finally, for morality, we found a very significantly lower probability to find positive (10%, posterior mean intercept = −2.15, 95% CI [−4.16, −1.21]) compared to negative morality characteristics. Not surprisingly, these data revealed a very large between‐participants variability, with a random‐intercept standard deviation of 1.29 (95% CI [0.05, 3.94]).

## GENERAL DISCUSSION

Previous studies focusing on social representation of autistic individuals on the one hand (e.g. Draaisma, 2009; Schell et al., 2024) and those building on the SCM on the other (e.g. Aubé et al., 2023; Boysen et al., 2020) appear to send an inconsistent message. The present research aimed to address this puzzle by proposing two improvements to the available literature. First, we analysed stereotypes associated with autistic individuals at the facet level rather than restricting the analysis to the Big Two dimensions. Second, we not only capitalized on classical trait attributions but also examined participants' spontaneous stereotypes.

Across two studies using different samples (i.e. student sample vs. general population sample), our results make two key contributions. First, the data suggest that examining stereotypes at the facet level is most informative because different facets lead to markedly distinct patterns of traits attribution as well as a different prevalence of spontaneous stereotypes. Second, these results confirm that a combination of classical trait attributions with more novel measures of spontaneous stereotypes allows us to capture a more nuanced rendition of the stereotypes that prevail about autistic people.

Considering classical trait attributions, our results help reconcile the apparent inconsistencies between previous SCM‐based studies and broader social representations of autism. Whereas SCM‐based studies generally stress the shortcomings of autistic people on the vertical dimension while pointing to their comparatively higher standing on the horizontal dimension (Aubé et al., 2023; Boysen et al., 2020; Canton et al., 2023; Görzig et al., 2020), more traditional work on representation of autism points to higher levels of intellectual skills but more modest levels of social skills (Draaisma, 2009; Wood & Freeth, 2016). As it turns out, our two studies deliver a slightly different message when looking at the dimension level. Whereas participants report societal perceptions of autistic people as slightly warmer than competent in our student sample (Study 1), this pattern reversed in the general population sample (Study 2), with slightly lower warmth than competence. These differences have two main implications. First, they confirm that societal instructions allow access to meaningful information regarding culturally shared representation, as societal stereotypes vary according to the cultural context of the targeted samples. Second, they reveal the presence of differences at the dimension level, with psychology students being less critical than the general population on the horizontal dimension. Interestingly, our integrative analysis showed that students' representations questioned the friendliness of autistic individuals to a lesser extent than the general population did, while simultaneously attributing slightly less morality. Clearly, the higher judgements of friendliness observed in this sample proved sufficient to create a warmer than competent judgement at the dimension level.

Specific characteristics of our two samples may account for these findings. Indeed, psychology students likely were more familiar with the concept of stereotypes and possibly more sensitive to issues of stigmatization and inclusion for such minority groups as autistic individuals. At the same time, the student sample reported lower levels of familiarity with autistic individuals than the respondents from the general population. This may be at least partly explained by the fact that, as psychology students were at the beginning of their training, they might be self‐aware of their lack of knowledge (Harris et al., 2020). Another explanation may rely on a possible selection bias within the general population sample, with an overrepresentation of people interested in psychological issues. Future work may want to deepen the analysis of disparities in stereotypical beliefs towards autistic individuals according to sample characteristics.

Having said this, the finding that our two studies show slightly different patterns at the dimension level nicely illustrates the dividends of taking facets into account when considering the stereotypes of autistic people. Indeed, at the facet level, our results do in fact send a consistent message in the two studies, with participants perceiving autistic individuals as low in friendliness but high in morality, which results in a moderate warmth score overall. A similar, though less pronounced, pattern can be observed for the competence dimension, with higher attributions of ability than of assertiveness.

Considering spontaneous stereotypes, our two studies emphasize the differences in centrality of the various facets. Specifically, in the case of autism, friendliness and ability emerged as the dominant facets, while morality and assertiveness seemed decidedly more peripheral. Furthermore, the characteristics spontaneously associated with autistic individuals were more positive for ability, but more negative for the other facets. This result is particularly telling with respect to the morality facet. Indeed, although classical trait measures suggest that perceivers attribute high morality to autistic individuals, a closer look at spontaneous stereotypes leads us to question this conclusion. Not only is morality far from central in respondents' spontaneous productions, but it is also mainly associated with negative characteristics. Such a pattern supports previous work emphasizing lay beliefs of violence in autistic individuals (Maras et al., 2015). This higher negative morality prevalence is also in line with efforts relying on the Spontaneous Stereotype Content Model (SSCM, Nicolas, 2023) showing that morality stereotypes are mainly negative.

Next to its implication in the context of autism, this discrepancy between trait attributions and spontaneous stereotypes is also interesting with respect to the research on stereotypes. It suggests that people responding to preselected traits do not tap into the same cognitive processes as spontaneously listing characteristics associated with a social group. Trait attributions may require individuals to draw on knowledge that allows them to position themselves, even if these elements are not always part and parcel of their stereotype. Conversely, spontaneous measures allow stereotypes to emerge in a less constrained manner and may thus give access to the central elements of people's stereotypes. Going back to negative morality, its highly diagnostic nature in a social context (Brambilla et al., 2013; Carrier et al., 2014; Mende‐Siedlecki et al., 2013) may partly explain its prevalence in spontaneous attributions. Overall, our data demonstrate the added value of using complementary measures to capture the full spectrum of stereotypes towards autistic individuals.

Considering that each evaluation facet is known to trigger distinct emotional and behavioural outcomes (Koch et al., 2024; see also exploratory analyses on OSF), the differences observed in our data at the facet level also send a very rich message regarding the kind of challenges faced by autistic individuals. Among the multiple obstacles experienced by autistic individuals are the difficulties associated with the development and maintenance of social relationships (Mazurek, 2014), with autistic individuals reporting unusually high levels of loneliness (Ee et al., 2019; Grace et al., 2022). Our results, and particularly those on the horizontal dimension, offer a possible explanation. Indeed, recent research suggests that each facet is associated with some exclusive phenomenology. Specifically, friendliness is primarily associated with pleasure and loyalty, whereas morality goes along with quiescence and the absence of threat (Carrier et al., 2014). In this regard, low perceptions of friendliness may hinder the possibility for autistic people to be seen as social agents, hence decreasing their opportunities to connect with others.

Hence, although the absence of threat may be a condition for the development of social relations, it does not on its own encourage non‐autistic individuals to interact and build relationships with autistic individuals. Moreover, our results regarding spontaneous stereotypes further suggest that even the premise that there must be no threat to develop social relations is called into question by the societal stereotypes about autistic individuals. The proportion of negative morality traits indeed aligns with beliefs suggesting that autistic individuals are inclined to criminal or violent behaviours (Im, 2016; Maras et al., 2015). This view runs against data indicating that autistic individuals are at higher risks of being the victim rather than the perpetrator (Cazalis et al., 2022; Gibbs et al., 2021; Gibbs et al., 2026). This means that, despite the absence of concrete evidence for these stereotypical beliefs, the belief that autistic individuals may be violent may favour their exclusion (Connolly & Beaver, 2016; Jones et al., 2022). Although verifying this conclusion falls outside the scope of the present work, future research should examine the predictive power of stereotypical views on behaviours towards autistic individuals. On a related note, stereotypes of low friendliness may also explain some misalignment within the kind of clinical support provided to autistic individuals. That is, beliefs that autistic individuals are asocial may create assumptions among clinicians that autistic individuals are not interested in initiating and maintaining social relationships, which may lead to an underestimation of their expectations on this front (Bennett et al., 2019; Darazsdi & Bialka, 2023).

As for the competence dimension and its facets of ability and assertiveness, they may help to understand the underemployment and wage disparities typically observed among autistic individuals (Bury et al., 2024; Cimera & Cowan, 2009). Indeed, knowing that assertiveness is a strong predictor of social status (Carrier et al., 2014; Yzerbyt et al., 2025), seeing autistic individuals as having higher levels of ability than of assertiveness may hinder their professional inclusion (Howlin, 2013) and could lead them to positions for which they are overqualified (Harvery et al., 2021). Having said this, this effect may be somewhat mitigated by the low prevalence of assertiveness in spontaneous productions, again underlying the importance to examining the predictive power of both trait attribution and spontaneous measurements of stereotypes. Furthermore, perceptions of high ability may lead to difficulties for autistic individuals to obtain the work accommodations they often need. Autistic individuals have been found to report feelings of helplessness, notably due to a sense of being misunderstood within the professional context (Davies et al., 2024; Lee et al., 2024; Smethurst et al., 2025). This situation may partly arise from the clash between stereotypical beliefs and professional realities of autistic individuals (e.g., fatigability) and may render access to workplace accommodations more difficult (Patton, 2019). Again, interesting as they may seem, these insights are strictly speculative, and future research may want to focus more specifically on approaching the barriers to workplace inclusion through the lens of stereotypes.

## CODA

Considering the apparent divergent images of autistic individuals in different strands of research, the present studies emphasize the importance of considering the facet level of social evaluation and spontaneous stereotypes when focusing on stereotypic views concerning autistic individuals. Our findings pave the way for future research aimed at disentangling the specific impact of each facet on emotional and behavioural responses. As we see it, future work is also needed to examine how trait attributions and spontaneous productions combine to shape the broader dynamics of stereotyping. These are two directions that stand high on our research agenda as they would both further our knowledge of the stereotyping process proper and shed light on the specific inclusion challenges that autistic individuals face in their everyday life.

## AUTHOR CONTRIBUTIONS

**Camille Sanrey:** Conceptualization; methodology; data curation; investigation; formal analysis; funding acquisition; visualization; writing – original draft; writing – review and editing; project administration. **Vincent Yzerbyt:** Writing – review and editing; supervision.

## CONFLICT OF INTEREST STATEMENT

The authors declare no conflicts of interest.

## Supporting information


**Table S1.** Means (standard deviations) and correlations [95% CI] between facets attribution (two‐items measures) in Study 1.
**Table S2.** Means (standard deviations) and correlations [95% CI] between facets attribution (sub‐sample excluding participants who failed at least one attention check).
**Table S3.** Means (standard deviations) and correlations [95% CI] between facets attribution with two‐items measures (Study 2).
**Table S4.** Means (standard deviations) and correlations [95% CI] between facets attribution (subsample excluding participants who failed at least one attention check).
**Figure S1.** Prevalence of each facet in spontaneous production according to valence among the sub‐sample excluding participants who failed at least one attention check (Study 1).
**Figure S2.** Prevalence of each facet in spontaneous production according to valence (subsample excluding participants who failed at least one attention check).

## Data Availability

All datasets, codebooks and analysis scripts can be found on the OSF page (Study 1: https://osf.io/t8qcm/?view_only=255c76e04e0049dcbbefeb00e7be042f; Study 2: https://osf.io/wp89y/?view_only=1abc9a9dc51a4f3d9fde50a1706d27be).
