# Supplementary material for: As warm as competent, really? On the importance of stereotype facets in the context of autism
Source: Br J Soc Psychol. 2026 Jul 8;65(3):e70112. doi: 10.1111/bjso.70112 (PMC13347110; doi:10.1111/bjso.70112)
Supplement: Supplementary file 1 — Table S1. Means (standard deviations) and correlations [95% CI] between facets attribution (two‐items measures) in Study 1. Table S2. Means (standard deviations) and correlations [95% CI] between facets attribution (sub‐sample excluding participants who failed at least one attention check). Table S3. Means (standard deviations) and correlations [95% CI] between facets attribution with two‐items measures (Study 2). Table S4. Means (standard deviations) and correlations [95% CI] between facets attribution (subsample excluding participants who failed at least one attention check). Figure S1. Prevalence of each facet in spontaneous production according to valence among the sub‐sample excluding participants who failed at least one attention check (Study 1). Figure S2. Prevalence of each facet in spontaneous production according to valence (subsample excluding participants who failed at least one attention check). [file BJSO-65-0-s001.docx]

**Supplementary Material**

This file contains information regarding robustness analyses for the two studies reported in the manuscript. Regarding the robustness analyses, three complementary analyses were done: (a) inclusion of the familiarity index as a covariate, (b) exclusion of participants who failed at least one attention check (descriptive statistics are reported in Table S1 for Study 1 and in Table S3 for Study 2), and (c) use of a two-item per facet measure (see Koch et al., 2024) instead of the initial three-item version (descriptive statistics are reported in Table S2 for Study 1, and in Table S4 for Study 2; prevalence in spontaneous stereotypes are represented in Figure S1 for Study 1 and in Figure S2 for Study 2).

**Study 1**

**Robustness analyses**

As a reminder, we ran three complementary analyses to analyze the robustness of our results. First, we reran the analyses with familiarity index as covariate. Second, we reran the analyses with two-items per facet index instead of the initial three-items index (see Koch et al., 2024 for the validation of this short measure). Third, we reran the analyses on both trait attribution and spontaneous stereotypes with a subsample (*N* = 391) excluding participants who failed at least one attention check (*n* = 106). Results are reported below and outline that our main results are robust to these three variations.

***With familiarity as covariate***

When familiarity is included as covariate, difference between warm and competence attribution became non-significant, *F*(1, 469) = 1.05, *p* = .207, partial *η*² = .003. Nevertheless, we still observe key differences at the facet level, *F*(2.71, 1270.22) = 132.03, *p* < .001, partial η² = .22. Bonferroni-corrected post-hoc comparisons still support our hypotheses: participants attributed significantly less friendliness than morality, *t*(469) = -18.96, *p* < .001, *d* = -0.85, and marginally less assertiveness than ability, *t*(469) = 3.32, *p* = .003^[[1]](#footnote-1)^, *d* = 0.15.

***With two-items per facet measure instead of the initial three-items version***

With two-items per facet measure (i.e., “warm” and “friendly” for friendliness; “honest” and “sincere” for morality; “skilled”^[[2]](#footnote-2)^ and “capable” for ability; and “confident” and “determined” for assertiveness), resulting in four-items per dimension, difference is still observed at the dimension level with higher attributions of warmth (M = 5.73, SD = 1.33) than competence (M = 4.95, SD = 1.48), *t*(1, 496) = 10.83, *p* < .001, *d* = 0.49. Furthermore, differences are still observed at the facet level (see Table S2 for descriptive statistics and correlations between facets), *F*(2.74, 1357.26) = 298.92, *p* < .001, partial η² = .38. Bonferroni-corrected post-hoc comparisons still support our hypotheses: participants attributed significantly less friendliness than morality, *t*(496) = -24.10, *p* < .001, *d* = -1.08, and less assertiveness than ability, *t*(496) = 11.82, *p* < .001, *d* = 0.53.

**Table S1.**

*Means (Standard Deviations) and correlations [95% CI] between facets attribution (two-items measures) in Study 1.*

|  | Mean (SD) | 1 | 2 | 3 | 4 |
| --- | --- | --- | --- | --- | --- |
| 1. Friendliness | 4.51 (1.86) | - |  |  |  |
| 2. Morality | 6.96 (1.64) | .16 ***  [.07-.24] | - |  |  |
| 3. Ability | 5.42 (1.80) | .11 *  [.02-.19] | .30 ***  [.22-.38] | - |  |
| 4. Assertiveness | 4.48 (1.65) | .18 ***  [.09-.26] | .34 ***  [.26-.42] | .46 ***  [.39-.53] | - |

*Note.* *** *p* < .001

***Without participants who failed at least one attention check***

**Trait attributions.** When analyses are made on the subsample excluding participants who failed at least one attention check, difference between warmth (*M* = 5.16, *SD* = 1.19) and competence (*M* = 4.95, *SD* = 1.48) attributions became non-significant, *t*(390) = 2.73, *p* = .007, *d* = 0.14, 95% CI [0.04, 0.24]. Nevertheless, we still observe our key differences at the facet level (see Table S3 for descriptive statistics and correlations between facets), *F*(2.02, 1042.67) = 904.30, *p* < .001, partial η² = .37. Bonferroni-corrected post-hoc comparisons still support our hypotheses: participants attributed significantly less friendliness than morality, *t*(390) = -25.47, *p* < .001, *d* = -1.14, and less assertiveness than ability, *t*(390) = 3.81, *p* < .001, *d* = 0.17.

**Table S2.**

*Means (Standard Deviations) and correlations [95% CI] between facets attribution (sub sample excluding participants who failed at least one attention check).*

|  | Mean (SD) | 1 | 2 | 3 | 4 |
| --- | --- | --- | --- | --- | --- |
| 1. Friendliness | 3.91 (1.63) | - |  |  |  |
| 2. Morality | 6.41 (1.43) | .20 ***  [.11-.30] | - |  |  |
| 3. Ability | 5.11 (1.73) | .12 *  [.02-.22] | .37 ***  [.28-.45] | - |  |
| 4. Assertiveness | 4.80 (1.63) | .21 ***  [.11-.30] | .41 ***  [.32-.49] | .55 ***  [.47-.61] | - |

*Note.* *** *p* < .001

**Spontaneous stereotypes.** A total of 1256 characteristics were produced within this subsample, of which 876 were coded in at least one facet of interest (69.75%). As the dictionaries allow for multiple coding (i.e., a single characteristic may be coded into more than one facet), we focused our analysis on pure characteristics–i.e., those coded in one facet only–yielding 822 coded characteristics (65.45% of overall production).

Among these 822 pure characteristics, 327 represent friendliness (26%), 45 morality (3.58%), 352 ability (28%), and 98 assertiveness (7.80%). Friendliness and ability still emerged as the most prevalent facets. Friendliness was significantly more prevalent than morality, χ²(1) = 213.77, *p* < .001, *w* = 0.76, and ability more prevalent than assertiveness, χ²(1) = 143.37, *p* < .001, *w* = 0.56. Characteristics were also coded for binary valence (i.e., positive vs. negative, see Figure S1 for a visual representation of results). In the spontaneous productions, friendliness (marginal), χ²(1) = 3.65, *p* = .056, *w* = 0.12, morality, χ²(1) = 15.36, *p* < .001, *w* = 0.59, as well as assertiveness, χ²(1) = 6, *p* = .014, *w* = 0.25, were significantly more often associated with negative than with positive characteristics. Conversely, ability was marginally more frequently associated with positive characteristics, χ²(1) = 3.68, *p* = .055, *w* = 0.10.

**Figure S1.**

*Prevalence of each facet in spontaneous production according to valence among the subsample excluding participants who failed at least one attention check (Study 1).*

*
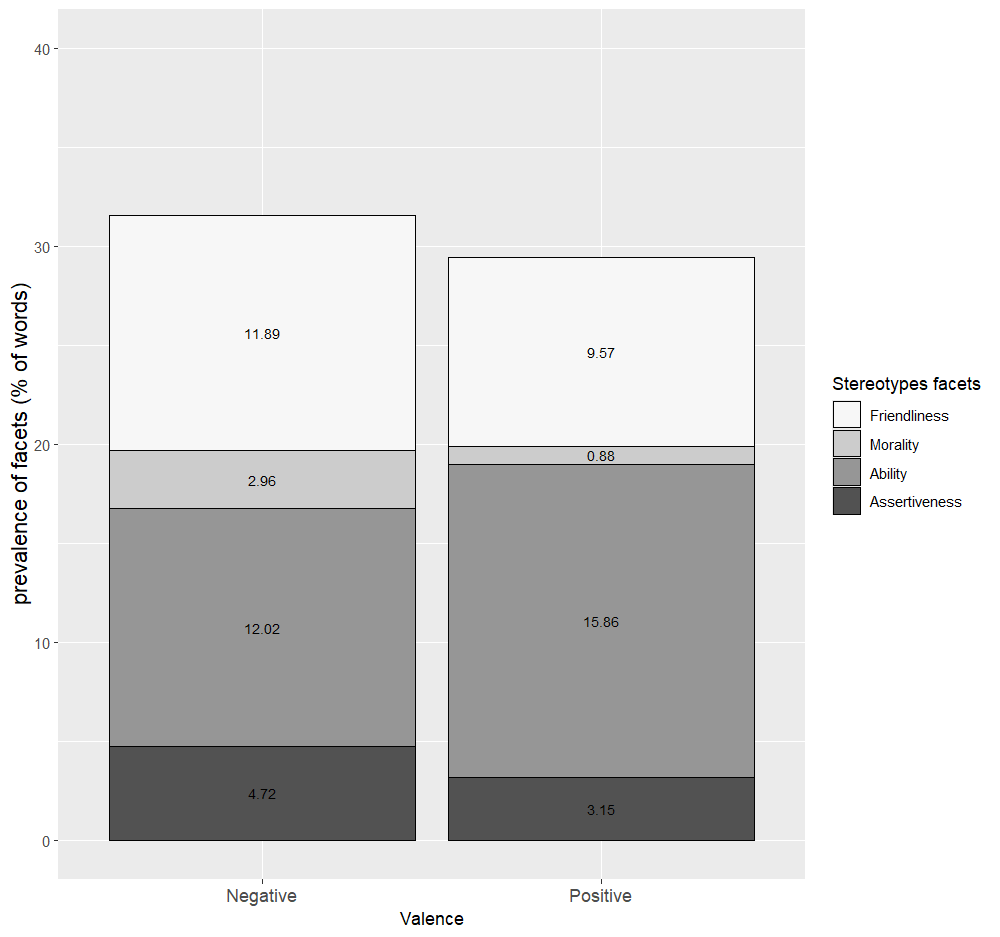
*

**Study 2**

**Robustness analyses**

We ran the same three complementary analyses as in Study 1: (a) including familiarity index as covariate. Second, we reran the analyses with two-items per facet index instead of the initial three-items index. Third, we reran the analyses on both trait attribution and spontaneous stereotypes with a subsample (*N* = 391) excluding participants who failed at least one attention check (*n* = 106). Results are reported below and outline that our main results are robust to these three variations.

***With familiarity as covariate***

When familiarity is included as covariate, difference between warm and competence attribution became non-significant, *F*(1, 636) = 6.41, *p* = .012, partial *η*² = .01. Nevertheless, we observe differences at the facet level, *F*(2.91, 1848.40) = 278.24, *p* < .001, partial η² = .30. Bonferroni-corrected post-hoc comparisons still partly support our hypotheses: participants attributed significantly less friendliness than morality, *t*(636) = -28.51, *p* < .001, *d* = -1.10, but not less assertiveness than ability, *t*(636) = 3.44, *p* = .003, *d* = 0.13 (*p* > .001).

***With two-items per facet measure instead of the initial three-items version***

With two-items per facet measure, difference is still observed at the dimension level with higher attributions of competence (*M* = 5.58, *SD* = 1.54) than warmth (*M* = 4.69, *SD* = 1.17), *t*(1, 666) = -15.63, *p* < .001, *d* = -0.61. Differences are still observed at the facet level (see Table S5 for descriptive statistics and correlations between facets), *F*(2.86, 1904.86) = 842.04, *p* < .001, partial η² = .56. Bonferroni-corrected post-hoc comparisons still partly support our hypotheses: participants attributed significantly less friendliness than morality, *t*(666) = -51.16, *p* < .001, *d* = -1.98, but not less assertiveness than ability, *t*(666) = -0.81, *p* = 1, *d* = -0.03.

**Table S3.**

*Means (Standard Deviations) and correlations [95% CI] between facets attribution with two-items measures (Study 2).*

|  | Mean (SD) | 1 | 2 | 3 | 4 |
| --- | --- | --- | --- | --- | --- |
| 1. Friendliness | 2.89 (1.25) | - |  |  |  |
| 2. Morality | 6.49 (1.68) | .26 ***  [.13-.27] | - |  |  |
| 3. Ability | 5.55 (1.59) | .23 ***  [.10-.25] | .34 ***  [.25-.39] | - |  |
| 4. Assertiveness | 5.61 (2.05) | .21 ***  [.13-.28] | .36 ***  [.21-.35] | .43 ***  [.31-.44] | - |

*Note.* *** *p* < .001

***Without participants who failed at least one attention check***

**Trait attributions.** When analyses are made on the sub sample excluding participants who failed at least one attention check (*N* = 540), competence attribution is still significantly higher (*M* = 5.03, *SD* = 1.42) than warmth attribution (*M* = 4.68, *SD* = 1.09), *t*(539) = -5.88, *p* < .001, *d* = -0.25, 95% CI [-0.34,-0.17]. We also still observe differences at the facet level (see Table S6 for descriptive statistics and correlations between facets), *F*(2.93, 1580.75) = 904.30, *p* < .001, partial η² = .63. Bonferroni-corrected post-hoc comparisons still support our hypotheses: participants attributed significantly less friendliness than morality, *t*(539) = -51.73, *p* < .001, *d* = -2.00, and less assertiveness than ability, *t*(539) = 6.41, *p* < .003, *d* = 0.25.

**Table S4.**

*Means (Standard Deviations) and correlations [95% CI] between facets attribution (sub sample excluding participants who failed at least one attention check).*

|  | Mean (SD) | 1 | 2 | 3 | 4 |
| --- | --- | --- | --- | --- | --- |
| 1. Friendliness | 2.72 (1.20) | - |  |  |  |
| 2. Morality | 6.65 (1.58) | .22 ***  [.14-.30] | - |  |  |
| 3. Ability | 5.25 (1.58) | .18 ***  [.10-.26] | .37 ***  [.29-.44] | - |  |
| 4. Assertiveness | 4.80 (1.74) | .25 ***  [.17-.33] | .36 ***  [.28-.43] | .51 ***  [.44-.57] | - |

*Note.* *** *p* < .001

**Spontaneous stereotypes.** A total of 1946 characteristics were produced within this subsample, of which 1253 were coded in at least one facet of interest (64.39%). Among these, 1204 are pure characteristics–i.e., coded in one facet only– (61.87% of overall production).

Among these 1204 pure characteristics, 617 represent friendliness (31.7%), 74 morality (3.80%), 384 ability (19.7%), and 129 assertiveness (6.63%). Friendliness and ability still emerged as the most prevalent facets. Friendliness was significantly more prevalent than morality, χ²(1) = 426.7, *p* < .001, *w* = 0.79, and ability more prevalent than assertiveness, χ²(1) = 126.75, *p* < .001, *w* = 0.50. Characteristics were also coded for binary valence (i.e., positive vs. negative, see Figure S1 for a visual representation of results). In the spontaneous productions, both friendliness, χ²(1) = 52.41, *p* < .001, *w* = 0.31, morality, χ²(1) = 38.48, *p* < .001, *w* = 0.73, and assertiveness, χ²(1) = 9.96, *p* = .001, *w* = 0.29 were significantly more often associated with negative than with positive characteristics. Conversely, ability was more frequently associated with positive characteristics, χ²(1) = 53.34, *p* < .001, *w* = 0.38.

**Figure S2.**

*Prevalence of each facet in spontaneous production according to valence (subsample excluding participants who failed at least one attention check).*

*
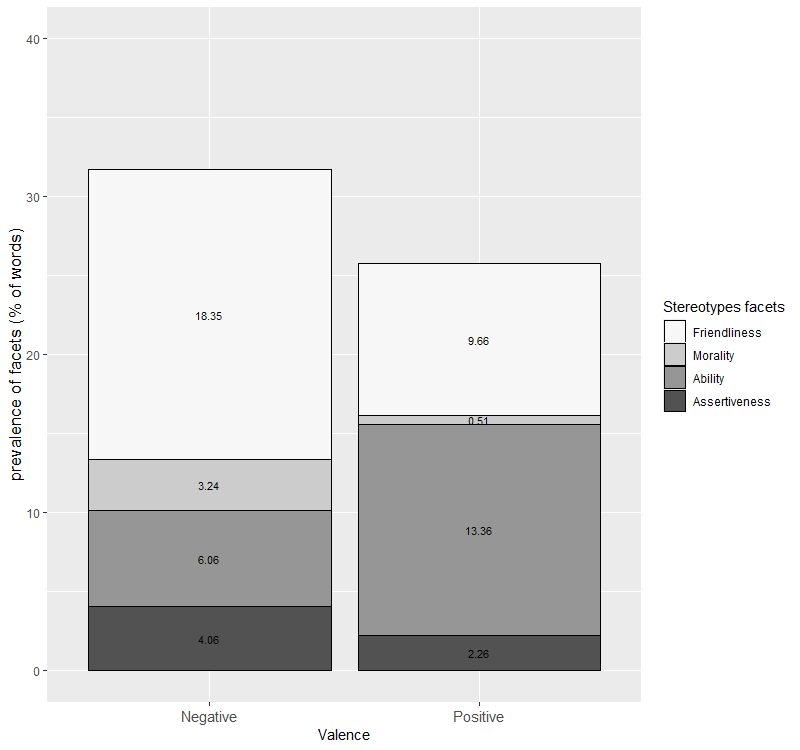
*

1. Remember that we used a more conservative Type I error level for traits attributions analyses. Accordingly, results are considered statistically significant at *p* < .004 (instead of the conventional .05). [↑](#footnote-ref-1)
2. Note that as we did not include this trait in our measures, leading us to use the most comparable trait: “intelligent” [↑](#footnote-ref-2)
